# Renal Sarcoidosis-like Reaction Induced by PD-1 Inhibitor Treatment in Non-Small Cell Lung Cancer: A Case Report and Literature Review

**DOI:** 10.3390/medicina59050991

**Published:** 2023-05-21

**Authors:** Sang-Don Park, Mee-Seon Kim, Man-Hoon Han, Yong-Jin Kim, Hee-Yeon Jung, Ji-Young Choi, Jang-Hee Cho, Sun-Hee Park, Chan-Duck Kim, Yong-Lim Kim, Jeong-Hoon Lim

**Affiliations:** 1 Division of Nephrology, Department of Internal Medicine, School of Medicine, Kyungpook National University, Daegu 41944, Republic of Korea; sd61644@gmail.com (S.-D.P.); hy-jung@knu.ac.kr (H.-Y.J.); jyss1002@hanmail.net (J.-Y.C.); jh-cho@knu.ac.kr (J.-H.C.); sh-park@knu.ac.kr (S.-H.P.); drcdkim@knu.ac.kr (C.-D.K.);; 2 Department of Pathology, School of Medicine, Kyungpook National University, Kyungpook National University Hospital, Daegu 41944, Republic of Korea; kimm23@naver.com (M.-S.K.); one-many@hanmail.net (M.-H.H.); yyjjkim@knu.ac.kr (Y.-J.K.)

**Keywords:** sarcoidosis-like reaction, immune checkpoint inhibitor, PD-1 inhibitor, kidney biopsy, lung cancer

## Abstract

Monoclonal antibodies directed against immune checkpoint proteins have been widely used to treat various cancers and have resulted in favorable clinical outcomes. Despite these beneficial properties, immune checkpoint inhibitors (ICIs) can induce side effects called immune-related adverse events, including sarcoidosis-like reactions (SLR) across multiple organs. Here, we report a case of renal SLR after ICI treatment, and we review the related literature. A 66-year-old Korean patient with non-small cell lung cancer was referred to the nephrology clinic for renal failure after the 14th pembrolizumab treatment dose. A renal biopsy revealed multiple epithelioid cell granulomas, with several lymphoid aggregates in the renal interstitium and a moderate degree of inflammatory cell infiltration in the tubulointerstitium. A moderate dose of steroid therapy was initiated, and the serum creatinine level partially recovered after four weeks of treatment. Judicious monitoring of renal SLR is, therefore, required during ICI therapy, and a timely diagnosis by renal biopsy and appropriate treatment are important.

## 1. Introduction

Immune checkpoints are molecules on immune cells that regulate the intensity and duration of immune responses. They prevent excessive immune activation and help maintain immune homeostasis [1]. Immune checkpoint inhibitors (ICIs) are promising anticancer agents that have enhanced the rate of sustained responses in cancer treatment. Cancer cells often employ mechanisms to evade immune recognition and attack [1]. They may express ligands, such as programmed death-ligand 1 (PD-L1), which bind to programmed cell death 1 (PD-1) on T-cells and inhibit their function. This interaction effectively dampens the immune response against the tumor.

ICIs are generally humanized monoclonal antibodies that inhibit down-regulatory immune pathways, such as PD-1 or its ligand (PD-L1), as well as cytotoxic T-lymphocyte antigen 4 (CTLA-4). By inhibiting PD-1 or CTLA-4, ICIs release the brakes on the immune system, T cells become activated, and antitumor immune responses are enhanced [2,3]. By administration of ICIs, T cells regain their effector function and cytotoxic activity against cancer cells; they infiltrate the tumor microenvironment, recognize cancer-specific antigens, and initiate immune-mediated cancer cell death [1]. As a consequence, ICIs lead to cancer regression and improve patient survival in many studies [4,5].

ICIs are clinically effective against various cancers, such as melanoma, non-small cell lung cancer, renal cell carcinoma, and urothelial cancer, and their scope of use is expanding [6,7,8,9,10]. Despite the beneficial properties of ICIs, they can also cause adverse autoimmune effects, including dermatitis, hepatitis, colitis, pneumonitis, and endocrinopathies [11], which are known as immune-related adverse events (irAEs). The incidence of irAEs has been reported to be between 15% and 90%, although the kidney has a low incidence of irAEs [11]. The incidence of renal irAEs is 2.2% in phase 2/3 clinical trials from a total of 3695 patients [12]. Most irAEs have shown organ-specific autoimmune or inflammatory features, and they can also cause systemic autoimmune disease, such as sarcoidosis [13]. This is called sarcoidosis-like reaction (SLR), and it is a rare, but important, irAE.

Many SLR cases occur within a few weeks following initiation of ICI treatment, and the use of CTLA-4 or a combination of ICI therapeutics causes SLR more frequently than PD-1 exposure alone [14]. The proposed mechanism by which ICIs promote SLR can be gleaned from the treatment-induced increase in lymphocyte counts and increased expression of T helper cell 1 (Th1)-associated markers [15,16]. The emergence of lymphocytes with increased expression of Th1-associated markers could potentially induce an SLR, as these cells are abundant in active sarcoidosis and are thought to be integral to the development of sarcoid granuloma [17]. Another mechanism appears to involve an increase in the number and function of Th17 cells [18]. Th17 cells are thought to play an integral role in the development of sarcoid granulomas and may promote the development of sarcoidosis-induced fibrosis [19]. In addition, there is evidence of a Th17 cell–T regulatory cell imbalance in sarcoidosis, with an increased Th17/T regulatory cell ratio both in the peripheral blood and bronchoalveolar lavage fluid of sarcoidosis patients [20].

The organs most affected by ICI-induced SLR are the lungs, lymph nodes, and skin [13,21], and the kidney is not an organ that is prone to occur SLR after ICI treatment. The most common renal histopathologic feature of acute kidney injury (AKI) caused by ICIs is acute interstitial nephritis, and ICIs occasionally cause immune complex glomerulonephritis, minimal change disease, focal segmental glomerulosclerosis, and thrombotic microangiopathy [12,22,23]. Here, we report a patient with non-small cell lung cancer who presented with AKI with renal SLR after anti-PD-1 antibody treatment.

## 2. Case Presentation

A 66-year-old Korean patient with non-small cell lung cancer was referred to the nephrology clinic because of deterioration of renal function. He was a 40 pack/year smoker and had idiopathic pulmonary fibrosis with a pattern of usual interstitial pneumonia. He had not taken any medication and was diagnosed with lung cancer through a left station 11 pulmonary lymph node biopsy in October 2019. A positron emission tomography scan demonstrated multiple uptakes in left lower lung, mediastinal lymph nodes, left pleura, and iliac bone, and there were no panda and lambda signs seen in sarcoidosis. He received four cycles of pemetrexed and cisplatin as first-line chemotherapy, but the cancer progressed.

**Figure 1:**
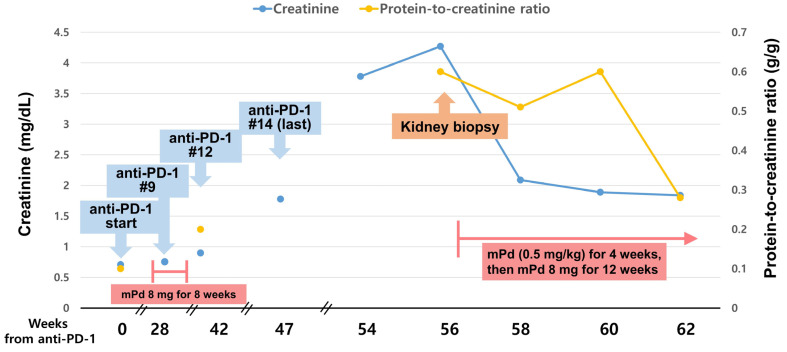
Clinical course after PD-1 inhibitor therapy.

The patient changed chemotherapy and received 200 mg pembrolizumab single-agent chemotherapy for lung cancer from February 2020. The treatment resulted in partial response, as the size of the mediastinal lymph nodes decreased, and the last (14th) dose was administered in March 2021. After the 9th pembrolizumab dose, the patient developed diffuse ground-glass opacity (GGO) lesions around the pulmonary fibrosis and newly enlarged neck lymph nodes around the left internal jugular vein less than 1 cm. The pulmonologist diagnosed ICI-related pneumonitis grade 1, and both GGO and neck lymphadenopathy were improved after treatment with 8 mg of methylprednisolone for eight weeks. The baseline serum creatinine level was 0.7 mg/dL, which increased to 1.8 mg/dL after the 14th dose of pembrolizumab (47 weeks of anti-PD-1 therapy; Figure 1). Since then, his condition deteriorated due to myalgia and diarrhea, and the anticancer treatment was hence discontinued. The patient’s diarrhea stopped, but generalized myalgia persisted, and he was hospitalized because his serum creatinine level had increased to 3.8 mg/dL. Prior to hospitalization, he had not taken any antibiotics, antirheumatic drugs, such as methotrexate and leflunomide, biologic agents, such as tumor necrosis factor (TNF)-α, or interleukin-2 receptor antagonists, proton pump inhibitors, and nonsteroidal anti-inflammatory drugs.

On physical examination, his initial blood pressure was 98/65 mmHg, with a pulse rate of 92 beats/min, and the axillary temperature was 36.3 °C. There were no other organ abnormalities, such as skin rash, eye, heart, and liver function. Abdominal computed tomography revealed a normal size and shape of both kidneys. Urinalysis revealed protein 1+ with a spot urine protein-to-creatinine ratio of 0.6 g/g, and there were no signs of microscopic hematuria. Laboratory findings revealed a white blood cell count of 10.43 × 10^3^/μL, and no eosinophils were detected. Both erythrocyte sedimentation rate and C-reactive protein levels were elevated to 29 mm/h (reference: 0–20.0 mm/h) and 2.15 mg/dL (reference: ≤0.3 mg/dL), respectively. Liver function tests and serum calcium level were within normal range. Other findings to identify glomerulonephritis, including anti-nuclear antibody, anti-neutrophil cytoplasmic antibody, anti-glomerular basement membrane (GBM) antibody, human immunodeficiency virus test, and anti-phospholipase A2 receptor antibody, were all negative. Both complement C3 and C4 levels were within the normal ranges.

**Figure 2:**
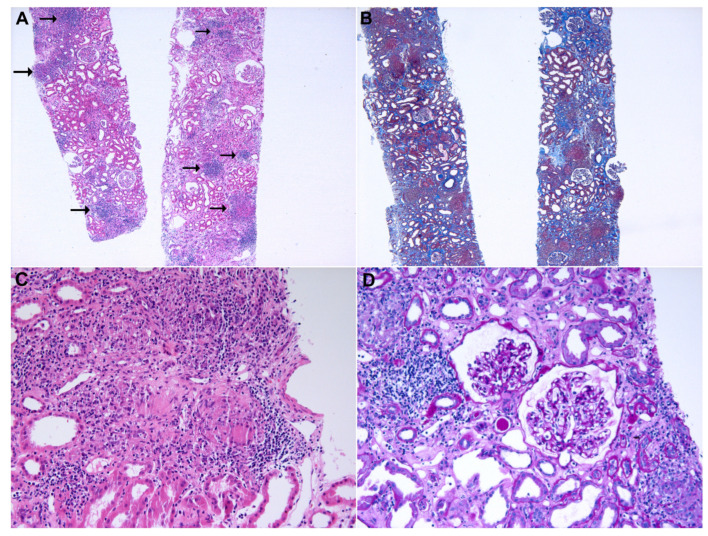
Histopathological findings of kidney biopsy. (**A**) Scattered discrete and uniform granulomas with lymphoid aggregates in the renal interstitium (arrows; hematoxylin and eosin; original magnification ×40). (**B**) A moderate degree of interstitial fibrosis and tubular atrophy (Masson’s trichrome; original magnification ×40). (**C**) Granulomas with tightly packed epithelioid cells and Langerhans cells with minimal lymphocytic cuffing (hematoxylin and eosin; original magnification ×200). (**D**) No significant pathological findings in the glomeruli (periodic acid-Schiff; original magnification ×200).

A renal biopsy was performed after 56 weeks of pembrolizumab treatment (Figure 2). Hematoxylin and eosin (H&E) staining revealed multiple epithelioid cell granulomas with several lymphoid aggregates in the renal interstitium. In addition, moderate degrees (25–50%) of interstitial fibrosis/tubular atrophy and inflammatory cell infiltration were observed in the tubulointerstitium. All 33 glomeruli had a normal appearance. Immunofluorescence was negative for IgG, IgA, IgM, C3, and fibrinogen levels. Electron microscopy revealed no electron-dense deposits and a normal GBM thickness.

To identify the cause of the granulomatous lesions, we performed Grocott’s methenamine silver staining to exclude fungal infection and acid-fast bacilli (AFB) staining to exclude tuberculosis, and the results were negative. The serum angiotensin-converting enzyme (ACE) level was elevated, at 78.3 U/mL (reference: 18.0–55.0 U/mL). Based on these results, the patient was diagnosed with pembrolizumab-induced renal SLR, and a moderate dose of steroid therapy (methylprednisolone at 0.5 mg/kg) was initiated. The serum creatinine level decreased to 1.8 mg/dL after four weeks of treatment, then methylprednisolone was tapered to 8 mg and used for 12 weeks. The use of PD-1 was discontinued given the patient’s general status, as well as the disease course of malignancy. After 19 months of cessation of ICI treatment, the cancer was stable without interval changes, and serum creatinine level remained at 1.6 mg/dL. ICI treatment did not restart because his cancer remained stable, and the patient did not want to take anticancer treatment.

## 3. Discussion

Here, we report a patient who developed AKI due to renal SLR with interstitial nephritis during PD-1 treatment. The patient’s baseline renal function was normal, but renal function gradually deteriorated after long-term administration of PD-1. ICIs caused severe renal dysfunction by occurrence of renal SLR with interstitial nephritis, but the renal function recovered after four weeks of methylprednisolone use. Therefore, if renal function abnormalities are observed during follow-up after administration of ICIs, it is necessary to confirm histopathological findings through kidney biopsy and to treat them early.

AKI that occurs during ICI therapy is mainly caused by acute tubulointerstitial nephritis and is readily overlooked. However, as in this case, the patient did not take other medication causing sarcoidosis, but renal SLR occurred after ICI administration. Histopathological findings are essential for the diagnosis of renal SLR. Immunohistochemical staining for CD4, CD8, and PD-L1 is useful for the diagnosis of SLR, and circulating CD4+ T-cell analysis may be suitable for treatment monitoring [12,14]. Therefore, histopathological diagnosis and assessment of chronicity by kidney biopsy will help not only diagnose renal SLR, but also determine the use of immunosuppressive agents, such as corticosteroids, which are potentially harmful agents, and this will determine whether to continue ICI administration [12].

SLR is a multisystemic disease and can lead to various organ dysfunctions. However, there are few data on SLR after ICI treatment, so many clinicians, including oncologists and nephrologists, are not interested in this rare disease. If the diagnosis is delayed, complications in multiple organs may result, so careful monitoring and early histological diagnosis of the involved organs are necessary for patients undergoing ICI therapy. To date, the treatment for SLR involves stopping ICI administration and the use of corticosteroids, and most patients have been shown to respond well [13]. However, most studies that reported SLR after ICI therapy have a small number of patients, and more large-scale studies are needed to confirm clinical course, prognosis, and treatment.

We reviewed and summarized previous studies of ICI-induced renal SLR (Table 1). The studies were all case reports because of the rarity of renal SLR and the time to onset of kidney disease after initiation of ICI therapy varies from two to thirty cycles. Nakatani et al. [24] reported a 68-year-old patient with gastric cancer who presented with renal SLR with acute interstitial nephritis after 30 cycles of PD-1 inhibitor. The initial serum creatinine was elevated to 3.8 mg/dL. After using methylprednisolone (1 mg/kg/day), the creatinine level was recovered to the baseline level. Then, PD-1 inhibitor was restarted, and there was no AKI recurrence. Person et al. [25] reported a 55-year-old patient with melanoma who presented with renal SLR and thrombotic microangiopathy-like lesion in a kidney biopsy. After two cycles of PD-1/CTLA-4 inhibitor treatment, severe AKI had occurred that requiring renal replacement therapy. High dose methylprednisolone (200 mg), mycophenolic acid, and TNF-α blockade (infliximab) were applied, but the response was poor, so AKI progressed to end-stage kidney disease. Thajudeen et al. [26] reported a 74-year-old patient with melanoma who presented with renal SLR with acute interstitial nephritis after three cycles of CTLA-4 inhibitor. The patient’s baseline creatinine was normal, but it increased to 2.2 mg/dL. After using high-dose prednisolone (60 mg/day), creatinine level was decreased to 1.4 mg/dL. Then, CTLA-4 inhibitor was restarted, and renal function remained stable. Izzedine et al. [27] reported a 60-year-old patient with melanoma who presented with renal SLR with acute interstitial nephritis after three cycles of CTLA-4 inhibitor. The patient’s baseline creatinine was normal, but it increased to 3.2 mg/dL. After using high-dose prednisolone (1 mg/kg/day) for four weeks, serum creatinine level was decreased to 1.3 mg/dL. Charkviani et al. [28] reported a patient in their early 60s with renal cell carcinoma who presented with renal SLR with acute interstitial nephritis after 10 weeks of PD-1/CTLA-4 inhibitor treatment. The patient had chronic kidney disease and baseline creatinine was 2.8 mg/dL, but it increased to 5.3 mg/dL. After using high-dose methylprednisolone (500 mg once, 80 mg/day for seven days, then tapered) for eight weeks, serum creatinine level was decreased to 2.1 mg/dL. Of the five cases, four patients exhibited a decrease in serum creatinine after treatment with high-dose steroids. However, one patient did not respond to immunosuppressive therapy, so caution is required. In addition, renal function was maintained in two cases wherein ICI therapy was restarted after the recovery of renal SLR. Permanent discontinuation of ICIs may affect overall patient survival. Therefore, in patients whose renal function has recovered, re-administration of ICI should not be routinely withheld because of concerns regarding AKI or SLR recurrence, and their reintroduction should be decided based on the risks and benefits for the patient in question.

## 4. Conclusions

In conclusion, ICI therapy can cause renal SLR in patients with malignancies. There are no specific symptoms, and it can start with mild proteinuria and gradual deterioration of renal function. Therefore, clinicians should keep in mind the possibility of renal SLR when administering ICI treatment. In case of doubt, it is recommended to check serum ACE levels and perform a histological confirmation by kidney biopsy.

## Figures and Tables

**Table 1:** Renal sarcoidosis-like reaction induced by immune checkpoint inhibitors.

Study	Age, Years	Sex	Immune Checkpoint Inhibitor	Primary Cancer	Onset (after ICI Cycle)	Histopathological Findings	Treatment	Outcome
Nakatani et al. [24]	68	F	Nivolumab (PD-1 inhibitor)	Gastric cancer	30th cycle	Granulomatous AIN	Steroid (mPd) 1.0 mg/kg/day (40 mg/day) for 7 days, 30 mg/day for 7 days, 20 mg/day for 7 days, then slowly tapered off	Resolved
Person et al. [25]	55	M	Nivolumab/Ipilimumab (PD-1/CTLA-4 inhibitor)	Melanoma	2nd cycle	Granulomatous AIN and thrombotic microangiopathy-like lesion	Steroid (mPd 200 mg once) Mycophenolic acid TNF-α blockade (infliximab)	Progression to ESKD
Thajudeen et al. [26]	74	M	Ipilimumab (CTLA-4 inhibitor)	Melanoma	3rd cycle	Granulomatous AIN	Steroid (Pd) 60 mg/day for 4 weeks, then tapered off for two weeks	Resolved
Izzedine et al. [27]	60	F	Ipilimumab (CTLA-4 inhibitor)	Melanoma	3rd cycle	Granulomatous AIN	Steroid (Pd) 1.0 mg/kg/day for four weeks, then tapered off quickly	Resolved
Charkviani et al. [28]	Early 60s	M	Nivolumab/Ipilimumab (PD-1/CTLA-4 inhibitor)	RCC	10 weeks	Granulomatous AIN	Steroid (mPd) 500 mg once, 80 mg/day for seven days, then tapered for eight weeks	Resolved

Abbreviations: ICI, immune checkpoint inhibitor; PD-1, programmed cell death 1; CTLA-4, cytotoxic T-lymphocyte antigen 4; AIN, acute interstitial nephritis; TNF, tumor necrosis factor; ESKD, end-stage kidney disease; Pd, prednisolone; mPd, methylprednisolone; RCC, renal cell carcinoma.

## Data Availability

The original contributions presented in this study are included in the article, and further inquiries can be directed to the corresponding author.
